# Intra-arterial chemoradiation for T3-4 oral cavity cancer: Treatment outcomes in comparison to oropharyngeal and hypopharyngeal carcinoma

**DOI:** 10.1186/1477-7819-6-2

**Published:** 2008-01-14

**Authors:** Ilana Doweck, K Thomas Robbins, Sandeep Samant, Francisco Vieira

**Affiliations:** 1 Department of Otolaryngology- Head and Neck Surgery, Carmel Medical Center, and Bruce Rappaport Faculty of Medicine, Technion – Israel Institute of Technology, Haifa, Israel; 2 Division of Otolaryngology-Head and Neck Surgery, Southern Illinois University School of Medicine, Springfield, IL, USA; 3 Department of Otolaryngology-Head and Neck Surgery, University of Tennessee, College of Medicine, Memphis, TN, USA

## Abstract

**Background:**

Surgery followed by radiotherapy is the standard of care for resectable locally advanced oral cavity squamous cell carcinoma (SCC). We report the treatment outcomes of patients with T3-T4 SCC of the oral cavity treated with chemoradiation, an alternative approach.

**Patients and methods:**

From a series of 240 patients with stage III-IV carcinoma of the upper aerodigestive tract who were treated consecutively according to the RADPLAT protocol, a subset analysis of 155 patients with T3-T4 SCC (Oral cavity SCC N = 22, oropharynx SCC N = 94 and hypopharynx SCC N = 39), was performed. The goal was to test the hypothesis that oral cavity SCC treated with chemoradiation has similar outcomes to the two comparison sites.

**Results:**

With a median follow-up of 58 months, local disease control was 69% and the overall survival was 37%. In comparison, local disease control for the oropharynx and hypopharynx groups was 86% and 79% respectively. The overall survival rate for the oropharyngeal and hypopharyngeal groups were 41% and 6% respectively

**Conclusion:**

Patients with locally advanced oral cavity cancer treated with the chemoradiation protocol RADPLAT have outcomes that are equal or better compared to patients with similar disease involving the oropharynx and hypopharynx

## Background

Chemoradiation has emerged as a viable option for patients with advanced head and neck cancer. Through meta-analyses and randomized trials, there is a growing body of evidence to indicate improved overall survival and organ preservation when compared to other treatment modalities [1-3]. However, there remains a paucity of data to determine whether there is a site-specific advantage for patients who present with advanced disease treated in this manner. Of particular interest are tumors arising in the oral cavity, a site where clinicians often show reluctance for treating patients with radiation, either alone or combined with chemotherapy. In contrast to this philosophy, we have followed the policy of offering patients with T3-4 oral cavity cancer chemoradiation, whether the disease is resectable or not [4]. Thus, over the interval of 7 years during which patients were treated with intra-arterial chemoradiation (RADPLAT), a substantial number with oral cavity cancer were enrolled.

We hypothesized that there were no significant differences in treatment outcomes based on site for patients receiving RADPLAT for T3-4 carcinoma of the oral cavity, oropharynx, and hypopharynx. The demonstration of equivalent efficacy for patients with oral cavity cancer would support the use of the RADPLAT protocol as an alternative to the current standard of care for advanced resectable oral cavity cancer: surgery and post-operative radiation therapy. The non-surgical approach may have the advantage of preserving function that frequently is associated with procedures like total and near-total glossectomy.

## Patients and methods

240 patients with Stage III-IV carcinoma of the head and neck were treated with the RADPLAT protocol at the University of Tennessee, Memphis, between 1993 and 2000. The data of these patients was previously reported in earlier studies, regarding analysis of distant metastasis [5] and predictors of local failure [6]. Within this prospectively collected database, we identified 155 patients with T3-4 carcinoma of the oral cavity (22 patients), oropharynx (94 patients) and hypopharynx (39 patients) to serve as the subjects for this analysis, to test the hypothesis that oral cavity carcinoma treated with RADPLAT has similar outcome to oropharyngeal and hypopharyngeal carcinoma. All patients in this subset analysis had advanced local disease, and surgery will be extremely mutilating. Nine of the patients (41%) with oral cavity carcinoma had T3 whereas 13 patients had T4 lesions (59%). Three patients (14%) had unresectable disease, and five patients had bone invasion (23%). All patients were entered onto an IRB-approved protocol and informed consent was obtained from all patients. All patients had biopsy proven squamous cell carcinoma. The demographics and tumor staging for each of the sites are outlined in Table 1.

**Table 1:** Patient and tumor characteristics based on site of disease.

Parameter	Oral Cavity	Oropharynx	Hypopharynx	P value
**Number**	22	94	39	
**Median Age (years)**	58.8	56.1	57.8	0.9
**Gender**				0.7
**Male:Female**	18:4	78:16	31:8	
**T classification**				0.34
**T3**	9 (41%)	45 (48%)	23 (59%)	
**T4**	13 (59%)	49 (52%)	16 (41%)	
**N classification**				0.54
**N0**	6 (27%)	25 (27%)	8 (20%)	
**N1**	3 (14%)	18 (19%)	7 (18%)	
**N2**	12 (54%)	44 (47%)	17 (44%)	
**N3**	1 (5%)	7 (7%)	7 (18%)	
**Stage**				0.31
**III**	3 (14%)	25 (28%)	12 (31%)	
**IV**	19 (86%)	69 (72%)	27 (69%)	

The RADPLAT protocol (4) included superselective, rapid, intra-arterial infusions of high dose cisplatin (150 mg/m2), which was delivered through a microcatheter. At the same time, sodium thiosulfate was given intra-venously to neutralize the systemic effects of cisplatin. The chemotherapy was delivered once each week over 3–4 consecutive weeks. Concomitant radiation therapy (2 Gy/fraction daily, 5 treatments/week over 7 weeks) was administered beginning on day 1 of the treatment, to a total dose of 70 Gy.

Patients were followed every week during the treatment protocol. Tumor response was determined during therapy by physical examination, and restaging was performed 2 months after radiation by means of criteria based on physical examination, computed tomography or magnetic resonance studies, and examination under anesthesia with biopsy of the tumor site. For patients with persistent lymphadenopathy, neck dissection was also performed at the same time.

We evaluated the following treatment outcomes of patients with oral cavity carcinoma and compared them to those with oropharyngeal and hypopharyngeal carcinoma:

1. Local failure, defined as histological evidence of carcinoma at the local site within 6 months following the completion of treatment (persistent disease), or histological evidence of carcinoma in the local site presenting after 6 months of follow-up (recurrent disease);

2. Regional failure, defined as recurrence in the cervical lymph nodes after completion of treatment;

3. Distant failure, defined as evidence of disease at distant sites without local or regional failure; and

4. Overall survival. 

Comparisons among the three sites were made for each of the four treatment outcomes.

The statistical analysis was done using JMP 4 for Windows (SAS Inc., NC.). Statistical analysis for all comparisons was done using the Chi square method. Estimates of local and regional disease control, and overall survival, at 5 years were done using the Kaplan-Meier method. The Log-rank test was used to determine the significance of the differences between the estimates for each subset. A Proportional Hazard Model was used to identify the parameters with the greatest effect on local control rate.

## Results

Among the total group of 155 patients, 22 had oral cavity cancer, 94 patients had oropharyngeal cancer, and 39 patients had hypopharyngeal cancer. The distribution of patients based on age, gender, T classification, N Classification, and stage, is shown in Table 1. There were no significant differences noted for each of these parameters. The mean age was 58 years (± 11 years, range 26–85.8 years). The median time for follow-up was 58 months (range 12–96 months), 46 months for oral cavity cancer patients, 58 months for patients with carcinoma of oropharynx, and 66 months for patients with hypopharynx primary. The differences are not significant (P = 0.12).

### Acute toxicity

Mucositis was the most common grade III-IV toxicity afflicting 49 patients (31%). This involved 8 patients (36%) with oral cavity cancer, 34 patients (36%) with oropharyngeal cancer, and 7 patients (18%) with hypopharyngeal cancer. There were no significant differences between the groups. Grade III-IV hematologic toxicity was observed in 17 patients (11%). There were no significant differences among the groups. Neurologic toxicity was the third most common grade III-IV acute toxicity, involving 8 patients (5%). Other categories of grade III-IV toxicities were: gastrointestinal – 4 patients; cardiac – 5 patients; circulatory – 1 patient; and otologic – 1 patient (Table 2)

**Table 2:** Distribution of Grade III-IV Toxicity based on Site of Disease

**Toxicity grade III & IV**	**Oral Cavity (n = 22)**	**Oropharynx (n = 94)**	**Hypopharynx (n = 39)**	**Total (n = 155)**
**Mucositis**	8 (36%)	34 (36%)	7 (18%)	49 (31%)
**Hematologic**	4 (18%)	11 (12%)	2 (5%)	17 (11%)
**Neurologic**	0	7 (7.5%)	1 (2.5%)	8 (5%)
**Gastrointestinal**	0	4 (4%)	0	4 (2.5%)
**Cardiac**	0	5 (5%)	0	4 (2.5%)
**Circulatory**	0	1 (1%)	0	1 (0.06%)
**Ototoxicity**	0	0	1 (2.5%)	1 (0.06%)
**Total Events**	12	62	11	85
**No. of patients**	11 (50%)	54 (57%)	10 (26%)	75 (48%)

10 patients had more than one episode of grade III-IV toxicity resulting in a total of 85 events among 75 patients.

### Local disease control

**Figure 1:**
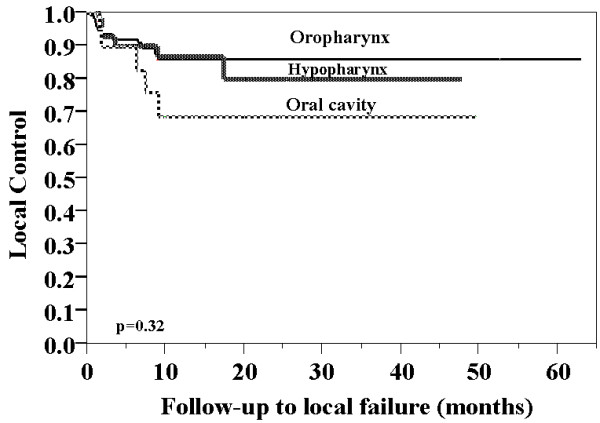
Local-control rate stratified by site of disease.

Based on the site of disease, the rate of local disease control for oral cavity was 17/22 (77%) compared to 83/94 (88%) for oropharynx and 33/39 (85%) for hypopharynx (X^2^, p = 0.42). The estimates of local disease control at 5 years using the Kaplan Meier method were 69% for oral cavity, 86% for oropharynx, and 79% for hypopharynx (figure 1). There were no significant differences among the 3 sites (Log-Rank test, p = 0.32). Using the Cox Proportional Hazard Model to determine which factors influenced the rate of local disease control, neither T classification or disease site were found to be significant.

### Regional disease control

Based on the site of disease, the rates of regional disease control were: oral cavity – 21/22 (97.5%); oropharynx – 91/94 (96.8%); and hypopharynx – 38/39 (99%).

### Distant metastases

Based on site of disease, the rates of disease failure initially occurring at distant sites were: oral cavity – 9%; oropharynx – 17%; hypopharynx – 36 % (p = 0.02).

### Survival

**Figure 2:**
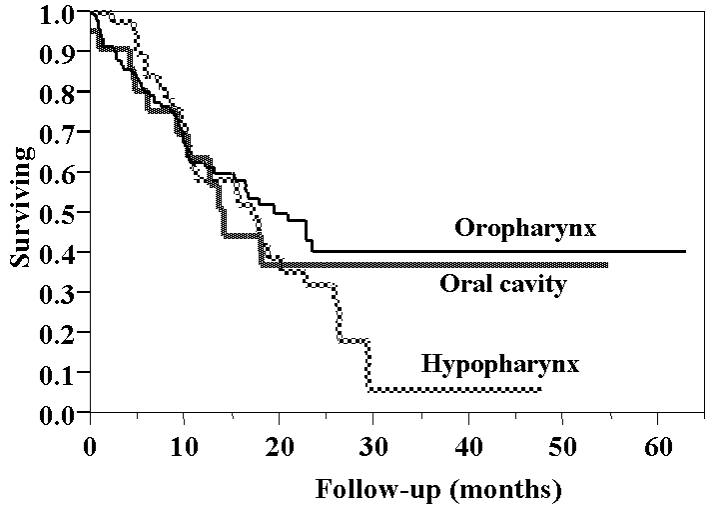
Overall survival stratified by site of disease.

At the time of analysis, the proportion of patients who remained alive according to site of disease was: oral cavity – 50%; oropharynx – 47%; hypopharynx – 27% (X^2^, p = 0.026). The rates of overall survival at 5 years using Kaplan Meier projections based on site of disease were: oral cavity – 37%; oropharynx – 41%; and hypopharynx – 6% (figure 2)

## Discussion

For patients who have resectable oral cavity disease, the current standard of care for T3-4 squamous cell carcinoma is surgery and postoperative radiation therapy. This treatment preference is accepted by radiation oncologists as well as surgeons and is most likely related to the biologic behavior of oral cavity carcinoma as well as the lower tolerance of oral cavity tissue for radiation therapy. Biologically, it is generally accepted by clinicians that squamous cell carcinoma of the oral cavity is more resistant to radiation therapy [7,8]. In addition, when radiation therapy is used as a primary modality, it is fraught with excessive toxicity, both acute and chronic. For acute problems, the major issue is mucositis. For example, radiation of the buccal mucosa often results in severe mucositis with ulcerations. Also, the lips are problematic to include in the radiation field because of the acute inflammation of the mucosa. The dominant chronic toxicity of radiation treatment to the oral cavity relates to osteoradionecrosis, many of which become clinically manifested years later. Although there were no recorded events in our series, longer follow-up may subsequently document this event. All patients in our series had dental evaluations prior to therapy and were managed according to the condition of the dentition, radiation fields, and patient compliance. Thus, the potential for such toxicity has influenced clinicians to treat patients with oral cavity carcinomas with primary surgery, even in the current era when chemoradiation is becoming the treatment of choice for other organ sites such as the larynx, oropharynx, and hypopharynx [2,3].

Although primary surgery for T3-4 oral cavity cancer remains the standard of care, a major disadvantage for patients undergoing this option is the associated functional morbidity. In particular, dysphagia remains a major challenge faced by patients undergoing surgery for oral cavity cancer because excessive soft and bony tissue removal is often necessary. Such procedures as total or near-total glossectomy, resection of the supra-hyoid musculature, and in some circumstances laryngectomy, have a major impact on quality of life. There clearly is a need to improve the treatment for T3-4 oral cavity cancer as this relates to morbidity as well as efficacy.

Although a number of chemoradiation studies have included patients with oral cavity tumors, the numbers of patients entered into such trials have typically lagged behind those with primary disease arising in other head and neck sites. Furthermore, among the chemoradiation trials reported, few have compared outcomes data specific for the oral cavity and thus it is difficult to know whether such protocols are effective for this site [9-15].

The 22 patients with T3-4 oral cavity carcinoma included in our analysis involved some patients, who had unresectable disease. More recently, these patients have been designated as T4b according to the AJCC staging system (2002) [16]. Patients in the oral cavity subset had a 69 % rate of local disease control, a rate that was not significantly different from the other 2 sites analyzed for comparison. Similarly, the projected overall survival rates at 5 years for patients with oral cavity tumors, was not significantly different when compared to patients with oropharyngeal tumors: 37% versus 41%. However, the 6% overall survival rate at 5 years observed for the patients with hypopharyngeal carcinoma was significantly less. This difference can be explained by the higher rate of distant metastases (36%) for hypopharyngeal cancer, a well-recognized characteristic of carcinomas arising in this site. We have previously reported that carcinomas arising in the hypopharynx treated with RADPLAT have the highest risk of distant failure, and when combined with the presence of nodal disease involving multiple levels, this rate approaches 60% [5].

A comparison of the results from our study with previously reported results of a treatment regimen consisting of accelerated radiation therapy shows a striking difference in the rate of local disease control. Fien *et al*., [7] treated 105 patients with oral tongue carcinoma using accelerated radiotherapy and found the rate of local disease control to be 45% for T3 disease and 0% for T4 disease. These results led the authors to recommend surgery with post-operative radiotherapy for advanced oral tongue carcinoma.

Mohr et al reported improved survival and loco-regional control in patients with T2-T4 patients with oral cavity and oropharyngeal carcinoma, treated with preoperative radio-chemotherapy followed by radical surgery, compared to patients with radical surgery alone. Patients with radical surgery alone had 31% loco-regional recurrence and 28% death, compared to 15.6% and 18.6%, respectively, in the subset of patients who were treated with pre-operative radio-chemotherapy [17]. Eckardt *et al*., found that in a protocol includjng Taxol^® ^and carboplatin given concomitantly with radiotherapy for 40 Gy, followed by surgery, 58% of the patients achieved a pathological complete response, and the 3 year overall survival rate was 84% [18].

Our study is in agreement with Balm *et al*., who reported treatment outcomes of 79 patients with unresectable carcinoma of the oral cavity, oropharynx, hypopharynx and larynx. The study included 20 patients with oral cavity carcinoma, and there was no difference in the outcome of the different sites regarding loco-regional control and survival [19].

The main purpose of our analysis was to document and compare the effects of the direct effects of the treatment such as disease control, survival, and toxicity. Functional outcomes such as swallowing and speech were not made in the same systematic fashion. However, we have previously reported on function outcomes for selected components of patients representing all sites treated with RADPLAT [20,21]. In future studies, it will be important to prospectively characterize such parameters specifically for the oral cavity site and in particular, compare this to patients with equivalent site-specific lesions who are treated surgically.

Our findings support the hypothesis that patients with oral cavity tumor are amendable to organ preservation protocols in which concurrent chemotherapy is given. The data indicates that there are no site-specific differences in loco-regional control for upper aerodigestive tract carcinomas treated with targeted chemoradiation (RADPLAT). Whether this feasibility is limited to protocols that employ the intra-arterial approach remains to be seen. The advantage of the intra-arterial technique is based on the blood supply to the oral cavity. Tumors arising in this site are amenable to selectively infusing the specific branches of the external carotid artery, particularly the lingual and facial arteries. Using contrast material and digital subtraction imaging during the capillary phase of the infusion, interventional radiologists are able to accurately select the dominant blood supply to the tumor bed.

## Conclusion

Patients with advanced oral cavity cancer treated with RADPLAT respond favorably to RADPLAT, and possibly other chemoradiation protocols. The effectiveness of the therapy is comparable to the results using the same protocol for oropharyngeal and hypopharyngeal cancer. It is likely that preservation of oral tissues such as the tongue can be achieved in the majority of cases. Whether this proves to preserve the function of the oral cavity such as mastication, deglution, and articulation, remains to be determined. Future trials of non-surgical treatment for this disease site should incorporate prospective analysis of such functions.

## Competing interests

The author(s) declare that they have no competing interests.

## Authors' contributions

ID: Contributed to concept and design, acquisition of data, analysis and interpretation of data, drafting and revising the manuscript KTR: contributed to concept and design, analysis and interpretation of data and revising the manuscript, SS: Helped in concept and design and revising the manuscript; FV helped in acquisition of data and revision of the manuscript. All authors read and approved final manuscript.
